# Prognostic value of ventricular diastolic dysfunction in patients with severe sepsis and septic shock

**DOI:** 10.5935/0103-507X.20150057

**Published:** 2015

**Authors:** Gustavo Rolando, Emilio Daniel Valenzuela Espinoza, Emelin Avid, Sebastián Welsh, Juan Del Pozo, Alejandro Risso Vazquez, Yanina Arzani, Fabio Daniel Masevicius, Arnaldo Dubin

**Affiliations:** 1 Instituto Médico de Alta Complejidad (IMAC) - Buenos Aires, Argentina.; 2 Sanatorio Otamendi - Buenos Aires, Argentina.

**Keywords:** Sepsis, Shock, septic, Echocardiography, Ventricular dysfunction, Aged

## Abstract

**Objectives:**

To evaluate the prevalence of myocardial dysfunction and its prognostic value in patients with severe sepsis and septic shock.

**Methods:**

Adult septic patients admitted to an intensive care unit were prospectively studied using transthoracic echocardiography within the first 48 hours after admission and thereafter on the 7^th^-10^th^ days. Echocardiographic variables of biventricular function, including the E/e' ratio, were compared between survivors and non-survivors.

**Results:**

A total of 99 echocardiograms (53 at admission and 46 between days 7 - 10) were performed on 53 patients with a mean age of 74 (SD 13) years. Systolic and diastolic dysfunction was present in 14 (26%) and 42 (83%) patients, respectively, and both types of dysfunction were present in 12 (23%) patients. The E/e' ratio, an index of diastolic dysfunction, was the best predictor of hospital mortality according to the area under the ROC curve (0.71) and was an independent predictor of outcome, as determined by multivariate analysis (OR = 1.36 [1.05 - 1.76], p = 0.02).

**Conclusion:**

In septic patients admitted to an intensive care unit, echocardiographic systolic dysfunction is not associated with increased mortality. In contrast, diastolic dysfunction is an independent predictor of outcome.

## Abstract (pt)

**Objetivo:**

Avaliar a prevalência de disfunção miocárdica e seu valor prognóstico em pacientes com sepse grave e choque séptico.

**Métodos:**

Pacientes sépticos adultos, admitidos em uma unidade de terapia intensiva, foram estudados de forma prospectiva por meio de ecocardiografia transtorácica dentro das primeiras 48 horas após sua admissão e, então, entre o sétimo e o décimo dias. As variáveis ecográficas de função biventricular, inclusive a relação E/e', foram comparadas entre sobreviventes e não sobreviventes.

**Resultados:**

Foi realizado um total de 99 ecocardiogramas (53 na admissão e 46 entre os dias 7 e 10) em 53 pacientes com média de idade de 74 anos (desvio padrão de 13 anos). Estava presente disfunção sistólica em 14 (26%); disfunção diastólica foi observada em 42 (83%) pacientes; e ambos os tipos de disfunção estavam presentes em 12 (23%) pacientes. A relação E/e', ou índice de disfunção diastólica, foi o melhor preditor de mortalidade hospitalar segundo a área sob a curva ROC (0,71) e se constituiu em um preditor independente do desfecho, conforme determinado pela análise multivariada (*odds ratio* - OR = 1,36 [1,05 - 1,76]; p = 0,02).

**Conclusão:**

Em pacientes sépticos admitidos em uma unidade de terapia intensiva, a disfunção sistólica determinada ecograficamente não se associa com aumento da mortalidade. Em contraste, a disfunção diastólica foi um preditor independente do desfecho.

## INTRODUCTION

Myocardial dysfunction is one of the mechanisms involved in the pathophysiology of septic shock. Septic myocardial dysfunction is usually defined as global (systolic and diastolic) but reversible biventricular dysfunction.^(1)^ An incidence ranging from 20 to 60% has been reported in the first 3 days after the onset of septic shock.^(2)^ Ventricular function generally returns to normal within 7 - 10 days.^(3)^

Left ventricular systolic function has been identified as a major prognostic factor for most cardiac diseases.^(4-7)^ The prognostic value of systolic dysfunction in sepsis, however, remains controversial.^(8)^ A study of 20 patients performed using radioisotopes and pulmonary artery catheterization has shown that systolic dysfunction and ventricular dilation occur in 50% of septic shock patients. Paradoxically, patients with systolic dysfunction have been demonstrated to have lower mortality.^(9,10)^ However, these findings have not been confirmed.^(11,12)^ A recent meta-analysis of 14 studies with a total of 887 patients has concluded that there are no significant differences in the ejection fraction or indexed ventricular dimensions between survivors and non-survivors.^(13)^ Another recent meta-analysis and systematic review that included seven studies with a total of 585 patients has found that a low ventricular ejection fraction is neither a sensitive nor a specific predictor of mortality in sepsis.^(14)^ Although it has been less studied, diastolic dysfunction is a common finding in septic patients, and it has recently been associated with a poor outcome.^(15-17)^

The aim of this study was to evaluate cardiac function using echocardiography in patients admitted to an intensive care unit (ICU) with severe sepsis and septic shock. Our objectives were to assess the following: 1) the prevalence of echocardiographic myocardial dysfunction; 2) the persistence of such abnormalities after the first 7 - 10 days following ICU admission; and 3) the prognostic value of left ventricular systolic and diastolic dysfunction for ICU mortality.

## METHODS

This is a single center, prospective observational cohort study conducted from July 2009 to April 2011 at a mixed medical-surgical ICU of a private hospital located in the inner metropolitan area of Buenos Aires, Argentina. This hospital has 200 beds and admits approximately 20,000 patients per year. The ICU has 28 beds and admits approximately 1300 patients per year. Was obtained approval from the Ethics Committee of *Instituto Médico de Alta Complejidad* (IMAC), resolution Nº 10609. Written informed consent was obtained from the next of kin of the patients. Anonymity was maintained by providing each patient with a unique identifier.

We included adult (aged 18 years or older) patients with a diagnosis of severe sepsis or septic shock^(18)^ who had been admitted to the ICU. We excluded patients with moderate-to-severe mitral and/or aortic valve disease and pre-existing severe impairment of the left or right ventricular ejection fraction (LVEF or RVEF, respectively) and those who had undergone myocardial revascularization surgery, received prosthetic valves, or had severe pulmonary hypertension.

The data were collected using a structured data collection form. We recorded patient demographic data, as well as cardiac echocardiography findings and patient outcomes. In addition, the Sequential Organ Failure Assessment (SOFA) score^(19)^ was calculated at the time of the first echocardiography, and the Acute Physiology and Chronic Health Evaluation (APACHE II) score^(20)^ was determined based on the first 24 h of the ICU stay.

All patients underwent transthoracic echocardiography examinations (Vivid 7, GE Medical Systems, Milwaukee, WI, USA). The first examination was performed within the first 48 hours of ICU admission, and the second was conducted between the 7^th^ and 10^th^ day of the ICU stay. A single cardiologist who specialized in echocardiography performed M-mode, 2-dimension and Doppler echocardiography. For these procedures, parasternal long- and short-axis, apical 4- and 2-chamber long-axis and subcostal views were utilized. Left ventricular end-diastolic and end-systolic volumes (LVEDV and LVESV, respectively) were measured using biplane modified Simpson's rule. Then, stroke volume (SV) and LVEF were calculated. The systolic and diastolic ventricular diameters and ejection and shortening fractions of both ventricles were measured. Ventricular diameters were adjusted for body surface area. The peak mitral and tricuspid inflow E and A velocity waves on pulsed-wave Doppler, E/A ratio, E-wave deceleration time, isovolumic relaxation time and color M-mode inflow velocity of propagation were measured from the apical four-chamber view. Tricuspid annular plane systolic excursion (TAPSE) was measured by M-mode in the apical four-chamber view.^(21)^ For diastolic assessment using tissue Doppler imaging (TDI), the sector width was narrowed stepwise to visualize the lateral myocardial wall to obtain good alignment between the wall and ultrasound beam and to reach a frame rate of at least 98 frames/second. At least 3 consecutive beats were recorded at end expiratory breathing in the non-ventilated patients. The images were digitally stored for off-line analysis. Thereafter, the e'/a' ratio and ventricular filling index E/e' ratio were calculated.

Systolic dysfunction was defined as an ejection fraction ≤ 50%. Diastolic function was categorized as follows:^(22,23)^ 1) normal: E/A ratio > 1, E wave deceleration time 120 - 220ms, and E/e' ratio < 11; 2) mild diastolic dysfunction: E/A ratio < 1, E wave deceleration time > 220ms, E/e' < 11, and e' wave < 5cm/s; 3) moderate diastolic dysfunction: E/A > 1, E wave deceleration time 120 - 220ms, and E/e' ratio > 11; and 4) severe diastolic dysfunction: E/A ratio > 2, E wave deceleration time < 120 ms, and E/e' ratio > 11. Diastolic function was dichotomized as normal or abnormal (normal versus mild, moderate and severe diastolic dysfunction).

### Data analysis

The distribution of continuous variables was explored using the Kolmogorov-Smirnov test. The first and the second set of echocardiographic measurements were compared with Student's *t* test or the Mann-Whitney *U* test. The chi square test or Fisher's exact test was used to assess qualitative variables. Odds ratios and 95% confidence interval (95%CI) were calculated. Logistic regression analysis was performed to determine the independent associations between the echocardiographic variables and outcome. Diastolic dysfunction parameters were included in multivariable regression analysis as continuous variables. The primary measure of outcome was ICU mortality. The predictive abilities of the echocardiographic variables were evaluated by receiver operating characteristic (ROC) analysis.

## RESULTS

Over the 21 month study period, 210 patients were deemed eligible for this study, 100 were excluded, and 50 were lost. Ultimately, 60 patients were included in the study. Seven patients were excluded because of difficulties with obtaining adequate echocardiogram images.

Twenty-nine (55%) of the patients were male, with a mean age of 74 (standard deviation - SD 13) years and a mean APACHE II score of 19 (SD 5). Thirty (57%) patients had a diagnosis of severe sepsis, and 23 (43%) had septic shock. A total of 58% of the patients were taking broad-spectrum antibiotics at admission, including 9 and 22 of the survivors and non-survivors, respectively (p = 0.61). Their clinical and epidemiological characteristics are shown in table 1.

**Table 1:** Clinical and epidemiological characteristics between survivors and non-survivors

	All	Survivors	Nonsurvivors	p value
	N = 53	N = 18	N = 35	
Age (years)	74 ± 13	71 ± 15	76 ± 12	0.30
Gender, male	29 (55)	31 (58)	22 (42)	0.19
Heart rate (beats/min)	86 ± 20	83 ± 15	87 ± 22	0.47
Mean arterial blood pressure (mmHg)	84 ± 13	85 ± 14	83 ± 12	0.59
APACHE II	19 ± 5	18 ± 4	20 ± 5	0.10
SOFA	7 ± 3	6 ± 3	8 ± 3	0.04
Surgical patients	10 (19)	6 (33)	4 (11)	0.07
Primary diagnosis of infection				
Pneumonia	34 (64)	12 (66)	22 (63)	1.00
Peritonitis	8 (15)	2 (11)	6 (17)	0.70
Other	10 (19)	2 (11)	8 (23)	0.46
Comorbidities				
Cerebrovascular accident	6 (11)	2 (11)	4 (11)	1.00
Arterial hypertension	22 (42)	9 (50)	13 (37)	0.39
Chronic cardiac insufficiency	12 (23)	2 (11)	10 (29)	0.18
COPD	10 (19)	3 (17)	7 (2O)	1.00
Arrhythmia	7 (13)	2 (11)	5 (14)	1.00
Chronic renal failure	2 (4)	0 (0)	2 (6)	0.54
ICU stay (days)	23 ± 19	29 ± 24	20 ± 14	0.10
Hospital stay (days)	23 ± 18	29 ± 23	22 ± 16	0.20
Mechanical ventilation				
Number of patients	45 (85)	15 (33)	30 (64)	0.82
Duration (days)	20 ± 16	23 ± 22	18 ± 12	0.94
Norepinephrine				
Number of patients	22 (42)	3 (14)	19 (86)	0.01
Dose (*µ*g/Kg/min)	0.28 ± 0.3	0.20 ± 0.05	0.29 ± 0.31	0.311
Days on vasopressors	5 ± 6	2 ± 5	7 ± 6	0.02

APACHE II - Acute Physiology and Chronic Health Evaluation; SOFA - Sequential Organ Failure Assessment; COPD - chronic obstructive pulmonary disease; ICU - intensive care unit. Results are expressed as a number (%) and the mean ± standard deviation.

Overall, 99 echocardiograms were performed. All patients received baseline echocardiogram within the first 48 hours after ICU admission. Seven patients died prior to undergoing echocardiogram between days 7-10.

There were no significant differences in the echocardiographic variables between the first and second measurements except for an increase in the left ventricular systolic volume and a decrease in the E/A ratio (Table 1S in electronic supplementary materials).

On admission, 14 patients (26%) had an LVEF ≤ 50%, 7 (13%) had an RVEF ≤ 50%, 42 (84%) had left ventricular diastolic dysfunction, 36 (83%) had right ventricular diastolic dysfunction, and 12 (23%) had systolic and diastolic dysfunction.

The echocardiographic findings on admission for the survivors and non-survivors are shown in table 2.

**Table 2:** Echocardiographic characteristics in survivors and nonsurvivors

	Survivors	Nonsurvivors	Odds ratio	p value
	N = 18	N = 35	(95%CI)*	
LV diastolic diameter (mm)	47 ± 7	48 ± 6	1.01 (0.92 - 1.10)	0.40
LV systolic diameter (mm)	31 ± 8	33 ± 7	1.04 (0.95 - 1.14)	0.10
LVDV (mL)	88 ± 60	87 ± 38	0.99 (0.99 - 1.01)	0.90
LVSV (mL)	39 ±39	42 ± 26	1.00 (0.98 - 1.02)	0.80
LV ejection fraction	59 ± 10	53 ± 11	0.95 (0.88 - 1.02)	0.06
S wave (cm/s)	11 ± 3	10 ± 5	0.94 (0.88 - 1.00)	0.80
RVDV (mL)	67 ± 29	63 ± 22	0.17 (0 - 45961)	0.60
RVSV (mL)	27 ± 17	27 ± 11	0.99 (0.97 - 1.01)	0.90
RV ejection fraction	62 ± 7	58 ± 10	0.99 (0.96 - 1.04)	0.10
TAPSE (mm)	23 ± 5	21 ± 4	0.94 (0.87 - 1.01)	0.20
LVDD	12 (29)	30 (71)	0.92 (0.80 - 1.05)	0.01
LV E deceleration time (m/s^2^)	211 ± 42	211 ± 72	7.50 (1.32 - 42.50)	0.10
LV e' (cm/s)	12 ± 4	13 ± 14	1 (0.99 - 1.00)	0.80
LV E/A ratio	1.0 ± 0.4	1.1 ± 0.5	1.78 (0.01 - 315)	0.40
LV E/e' ratio	6.6 ± 2.5	8.8 ± 2.9	1.71 (0.43 - 6.93)	0.01
RVDD	9 (24)	29 (76)	1.36 (1.06 - 1.75)	0.02
LV E deceleration time (m/s^2^)	214 ± 64	194 ± 67	0.99 (0.98 - 1.00)	0.30
LV e' (cm/s)	13 ± 3	14 ± 8	27.16 (0.00 - 53.30)	0.51
LV E/A ratio	0.97 ± 0.3	1.11 ± 0.4	3.40 (0.55 - 21.10)	0.18
LV E/e' ratio	3.8 ± 1.2	4.3 ± 1.8	1.21 (0.83 - 1.78)	0.32
LVSDD	2 (11)	10 (29)	3.20 (0.62 - 16.54)	0.16

LV - left ventricle; RV - right ventricle; LVDV - left ventricular diastolic volume; LVSV - left ventricular systolic volume; TDI - tissue Doppler imaging; RVDV - right ventricular diastolic volume; RVSV - right ventricular systolic volume; TAPSE - tricuspid annular systolic plane excursion; LVDD - left ventricular diastolic dysfunction; LVSD - left ventricular systolic dysfunction; RVDD -right ventricular diastolic dysfunction; LVSDD - left ventricular systolic and diastolic dysfunction;

* 95% confidence interval. Results are expressed as a number (%) and the mean ± standard deviation.

The patients with right ventricular diastolic dysfunction had a longer duration of mechanical ventilation compared to those without ventricular dysfunction (20 ± 16 versus 11 ± 5 days, p = 0.01). The patients with left and right ventricular diastolic dysfunction were older (Table 2S in electronic supplementary materials).

A total of 85% of the patients were on mechanical ventilation. The tidal volume (ml/Kg) means were 7.6 ± 1 and 7.5 ± 1, the positive end-expiratory pressure (PEEP) means were 6 ± 2 and 7 ± 3cmH_2_O, and the PO_2_/FiO_2_ ratios (partial pressure of oxygen/inspiratory oxygen fraction) were 280 ± 80 and 250 ± 90mmHg for the survivors and non-survivors, respectively. None of these differences were statistically significant.

Overall, the ICU mortality rate was 66%. Multivariate regression analysis of ICU mortality showed that only the E/e' ratio and SOFA score were independent predictors of mortality (Table 3). The areas under the ROC curves of the SOFA score and E/e' ratio for predicting lCU mortality were 0.67 (95%CI 0.52 - 0.82) and 0.71 (95%CI 0.56 - 0.86), respectively. The pooled sensitivities and specificities of E/e' ratios > 11.0 and > 8.0 for predicting ICU mortality were 50% (95%CI 0.27 - 0.73) and 94% (95%CI 0.79 - 0.99) and 54% (95%CI 0.37 - 0.70) and 77% (95%CI 0.52 - 0.93), respectively.

**Table 3:** Multivariate logistic regression analysis of intensive care unit mortality

	Odds ratio (95%CI)	Wald	p value
E/e' ratio	1.36 (1.05 - 1.76)	5.74	0.02
SOFA	1.28 (0.99 - 1.65)	3.81	0.05

95%CI - 95% confidence interval; SOFA - Sequential Organ Failure Assessment.

## DISCUSSION

The main finding of our study was that old septic patients had a high prevalence of diastolic dysfunction. This dysfunction persisted over the course of 7 - 10 days. Moreover, it was associated with a poor outcome, and the E/e' ratio was the best echocardiographic parameter for predicting mortality. Finally, systolic dysfunction was not associated with survival.

We found prevalence rates of 84% and 83% for left and right ventricular diastolic dysfunction, respectively, in the group of septic patients. These figures are higher than those previously described in other series of septic patients, for example, incidences of left ventricular diastolic dysfunction of 37% and 38% have been reported.^(11,17)^ Because diastolic dysfunction is more frequent in older patients,^(24)^ the discrepancies in these findings might be related to a difference in the mean age of our patients (74 years, SD 13) compared to other studies (65 years, SD 15)^(11)^ and (61 years, SD 19).^(17)^ Moreover, we found that diastolic dysfunction was associated with increased mortality. This relationship was still present after adjusting for age and the SOFA and APACHE scores. The finding of an association of decreased ventricular compliance with poor outcome may be consistent with the results of a previous study demonstrating a lack of ventricular dilation in non-survivors with septic shock.^(10)^

Although we used a thorough approach for the assessment of diastolic function, only the E/e' ratio was independently related to outcome. This ratio represents the relationship between early mitral inflow velocity and mitral annulus movement. Therefore, it is an estimate of cardiac filling pressure. Accordingly, an observational study of patients with septic shock has found a strong correlation between pulmonary artery wedge pressure and the E/e' ratio.^(25)^ An elevated E/e' ratio indicates poor ventricular compliance. Because this ratio was found to be the only independent predictor of mortality in this study, it may be a better prognostic indicator than the other variables associated with diastolic failure. An alteration in the E/e' ratio has been described previously in patients with septic shock.^(22)^ Although one study found that the E/e' ratio is an independent predictor of mortality,^(17)^ another investigation failed to find this association.^(11)^ Nevertheless, the upper limit of the E/e' ratio in septic shock has not been fully defined because it can change according to the intravascular volume, whether it is measured before or after resuscitation, and with the use of mechanical ventilation as well as inotropic drugs. Our findings suggest that an E/e' ratio of greater than 8.0 might be a predictor of mortality. Nevertheless, the current E/e' ratio reference limits are based on patients with cardiac disease.^(22,23)^

Left and right systolic dysfunction was present in 26% and 13% of the patients, respectively. As previously described, systolic dysfunction was not related to ICU mortality.^(8,13,14)^ Similarly, changes in ventricular diameter or volume did not affect the outcome.

In addition to the ejection fraction, the S wave, as determined by spectral TDI, can be used to assess systolic function. The S wave may more accurately reflect myocardial contractility than the ejection fraction because it is less dependent on cardiac filling pressure.^(26,27)^ Weng et al. have found that an S wave > 9cm/s is predictive of mortality in septic patients.^(28)^ In our study, we did not find differences in the S wave value between the survivors and non-survivors.

Left ventricular diastolic dysfunction, as assessed according to the E/e' ratio, is a prognostic indicator of mortality in septic patients,^(15-17,29)^ and it is highly prevalent in older patients, as shown in our study. However, there are no therapeutic strategies available for ameliorating this dysfunction. Moreover, methodological differences in evaluation of the E/e' ratio may exist among studies, such as differences in the site of tissue Doppler analysis, the assessment of a single beat or averaged beats, the respiratory cycle and the influence of mechanical ventilation.^(17,28,30)^ The reliability of TDI for measurement of the E/e' ratio for assessing diastolic dysfunction at the bedside of critically ill patients was not addressed in this study. Additional clinical studies are required to examine mortality outcomes if lusitropic or negative fluid balance is used to improve diastolic dysfunction in septic critically ill patients.

An important strength of this study is that the echocardiographic examination was repeated after a period of 7 - 10 days. The lack of resolution of the alterations in cardiac function might mean that these abnormalities were premorbid conditions. However, our study has some limitations: our sample size was small, and the study was performed at a single center. Furthermore, prior ventricular function was unknown. Thus, it was not possible to discern whether alterations in diastolic function preceded or were a consequence of sepsis, However, the presence of diastolic dysfunction in patients appears to indicate an increased risk of death.

## CONCLUSION

In conclusion, our prospective observational study has evaluated cardiac function via echocardiography in septic intensive care unit patients and has found that left ventricular diastolic dysfunction, as assessed according to the E/e' ratio, is highly prevalent in old patients and is an independent predictor of mortality. Further investigations of the prognostic value and clinical usefulness of early echocardiography for septic patients appear warranted.
