# Supplementary material for: Prognostic value of ventricular diastolic dysfunction in patients with severe sepsis and septic shock
Source: Rev Bras Ter Intensiva. 2015 Oct-Dec;27(4):333–9. doi: 10.5935/0103-507X.20150057 (PMC4738818; doi:10.5935/0103-507X.20150057)
Supplement: Supplementary file 1 [file rbti-27-04-0333-suppl01.pdf]

Prognostic value of ventricular diastolic dysfunction in patients with severe sepsis and septic shock

Valor prognóstico da disfunção ventricular diastólica em pacientes com sepse grave e choque séptico

Gustavo Rolando[1,2], Emilio Daniel Valenzuela Espinoza[2], Emelin Avid[2], Sebastián Welsh[2], Juan Del Pozo[2], Alejandro Risso Vazquez[2], Yanina Arzani[2], Fabio Daniel Masevicius[2], Arnaldo Dubin[2]

Table 1S - Comparison of the echocardiographic variables between the first and second measurements

	At admission N = 53	7-10 days N = 46	p value
LV diastolic diameter (mm)	48 ± 6	47 ± 6	0.60
LV systolic diameter (mm)	32 ± 7	31 ± 6	0.08
LVDV (mL)	88 ± 47	87 ± 31	0.85
LVSV (mL)	26 ±14	38 ± 21	0.01
LV shortening fraction	33 ± 9	35 ± 8	0.09
LV ejection fraction	56 ± 11	57 ±10	0.43
S wave (cm/s)	9.9 ± 3.0	10.6 ± 3.0	0.20
RVDV (mL)	64 ± 24	64 ± 21	0.89
RVSV (mL)	26 ± 14	25 ± 11	0.43
RV ejection fraction	59 ± 9	61 ± 10	0.29
TAPSE (mm)	22 ± 5	22 ± 4	0.92
LVDD	42 (84)	40 (88)	0.36
E wave deceleration time (m/s^2)	213 ± 66	230 ± 66	0.14
e' wave (cm/s)	12.8 ± 12.0	10.9 ± 3.0	0.33
E/A ratio	1.0 ± 0.5	0.9 ± 0.4	0.24
E/e' ratio	8.1 ± 3.1	7.5 ± 2.7	0.25
RVDD	36 (77)	42 (95)	0.25
E wave deceleration time (m/s^2)	205 ± 67	227 ±	0.08
e' wave (cm/s)	14.0 ± 7	13.5 ± 7	0.75
E/A ratio	1.0 ± 0.3	0.8 ± 0.3	0.04
E/e' ratio	4.0 ± 1.6	4.0 ± 1.7	0.97

LV - left ventricle; LVDV - left ventricular diastolic volume; LVSV - left ventricular systolic volume; RVDV - right ventricular diastolic volume; RVSV - right ventricular systolic volume; RV - right ventricle; TAPSE - tricuspid annular systolic plane excursion; LVDD - left ventricular diastolic dysfunction; LVSD - left ventricular systolic dysfunction; RVDD - right ventricular diastolic dysfunction. Results are expressed as a number (%) and the mean ± standard deviation.

Table 2S - Clinical and epidemiological characteristics of systolic and diastolic dysfunction in both ventricles

	Systolic dysfunction						Diastolic dysfunction					
	Left ventricle			Right ventricle			Left ventricle			Right ventricle		
	Yes	No	p value	Yes	No	p value	Yes	No	p value	Yes	No	p value
N (%)	14 (26)	39 (74)		7 (13)	46 (87)		42 (84)	8 (16)		36 (83)	11 (17)	
Age (years)	74 ± 10	74 ± 15	0.89	73 ± 16	74 ± 13	0.83	77 ± 12	58 ± 14	0.01	77 ± 11	65 ± 17	0.01
APACHE II	22 ± 6	18 ± 4	0.03	20 ± 5	19 ± 5	0.84	19 ± 5	17 ± 4	0.26	20 ± 6	17 ± 4	0.08
SOFA	8 ± 3	7 ± 3	0.13	7 ± 2	7 ± 3	0.78	7 ± 3	6 ± 3	0.53	7 ± 3	6 ± 3	0.11
MV (days)	16 ± 10	20 ± 17	0.46	19 ± 16	20 ± 17	0.94	19 ± 15	25 ± 24	0.55	20 ± 16	11 ± 5	0.01
ICU stay (days)	19 ± 18	24 ± 18	0.36	15 ± 9	24 ± 19	0.21	22 ± 17	22 ± 17	0.19	22 ± 18	16 ± 6	0.29
Hospital stay (days)	20 ± 18	25 ± 18	0.35	15 ± 9	25 ± 19	0.16	24 ± 22	24 ± 22	0.66	22 ± 17	21 ± 14	0.93

APACHE II - Acute Physiology and Chronic Health Evaluation; SOFA - Sequential Organ Failure Assessment; MV - mechanical ventilation; ICU - intensive care unit. Student's *t* test or Mann Whitney test was used as appropriate. Results are expressed as the mean ± standard deviation.
